# Synthesis of Chromium Carbide Nanopowders by Microwave Heating and Their Composition and Microstructure Change under Gamma Ray Irradiation

**DOI:** 10.3390/molecules24010016

**Published:** 2018-12-20

**Authors:** Kai Jin, Yuanbo Jia, Zhiwei Zhao, Weiqiang Song, Shun Wang, Chunlong Guan

**Affiliations:** College of Materials Science and Engineering, Henan University of Technology, Henan 450001, China; 13298191127@163.com (K.J.); 15649856931@163.com (Y.J.); weiqiang_song@haut.edu.cn (W.S.); shun_wang@haut.edu.cn (S.W.); chunlong_guan@haut.edu.cn (C.G.)

**Keywords:** microwave heating, gamma irradiation, HRTEM, microstructure, chromium carbide

## Abstract

Chromium carbide nanopowders were synthesized by mechanical alloying-assisted microwave heating. The effect of gamma irradiation on phase composition and microstructure of chromium carbide nanopowders synthesized by the microwave heating method was analyzed. The samples were characterized by X-ray diffractometry (XRD), X-ray photoelectron spectroscopy (XPS), scanning electron microscopy (SEM), transmission electron microscopy (TEM), and high-resolution transmission electron microscopy (HRTEM) techniques. The results showed that well-dispersed chromium carbide nanopowders can be synthesized by maintaining the temperature at 1000 °C for 1 h. Gamma ray irradiation had an important effect on the microstructure of chromium carbide nanopowders. The interplanar spacings of chromium carbide (110) crystal faces before and after gamma ray irradiation were 0.3725 nm and 0.3824 nm, respectively. The crystal structure of chromium carbide was changed by gamma ray irradiation. Gamma ray irradiation can also increase the binding energy of chromium carbide, which is beneficial to improve the thermal stability and mechanical properties of chromium carbide at high temperature.

## 1. Introduction

The transition metal carbides have attractive mechanical properties (e.g., excellent strength, hardness, anti-erosion qualities), outstanding corrosion properties, positive temperature coefficients of resistivity, and permanent non-magnetizability [1]. Chromium carbide (Cr_3_C_2_), as a typical representative, demonstrates excellent properties, including high melting point (1810 °C), excellent compressive strength (4.1 GPa [2]), high hardness (Hv, 18 GPa [3]), low density (6.68 g/cm^3^ [4]) and good resistance to oxidation, corrosion, and wear. As a result, Cr_3_C_2_ has been widely used in a variety of industrial applications, such as rocket nozzles, shaft seals, shaft bearings, cutting tools, and anticorrosive coating [5,6,7].

Until now, there have been a number of methods for the preparation of chromium carbides, including direct element reaction, gas reduction-carburization, and conventional carbothermal reduction [8,9,10]. In the above-mentioned methods, the most commonly used method for synthesizing Cr_3_C_2_ is the carbothermal reduction method using micron-sized chromium oxide and carbon powders as raw materials. However, the process usually requires a high reaction temperature (>1400 °C) and a long reaction time (20–40 h) [11].

Mechanical alloying (MA) has been considered a powerful and practical process for the fabrication of several advanced materials including solid solutions, intermetallic phases, nano-structured materials, and amorphous alloys [12,13]. Microwave heating has become an interesting method for the synthesis of ceramic materials [14]. It generates heat within the material, which then spreads in the entire volume, and can save energy and time [15]. In this paper, Cr_3_C_2_ nanopowders were synthesized by mechanical alloying-assisted microwave heating using micron-sized Cr_2_O_3_ and nano-sized carbon black as raw materials. The direct and fast heating enhanced by the presence of carbon, led to the synthesis of pure carbides in a reduced time, with very crystalline, fine particles presenting excellent and interesting morphology [16].

Nowadays, irradiation (with electrons, ions, gamma rays, etc.) is considered an emerging innovative, valuable tool for modifying the structure of nanomaterials [17,18]. Gamma ray irradiation with different strengths is a powerful physical treatment that can produce structural ordering and disordering of nanomaterials [19,20]. Kleut et al. [21] studied the gamma irradiation effects on the structure of single-wall carbon nanotubes (SWCNT). Their results showed that gamma irradiation caused SWCNT covalent functionalization, and the degree of disorder in the carbon nanotube structure correlated with the irradiation dose. Naim et al. [22] reported the effect of gamma irradiation on the mechanical properties of a PVC/ZnO polymer nanocomposite. The results showed that gamma irradiation resulted in a detectable decrease of the elastic modulus for pure and low-weight ratio ZnO nanoparticles. However, the irradiation dose did not have any effects on the elastic modulus of nanoparticles with a wt% ratio of 10.

Little attention has been paid to the comparison between the effects of irradiation and those of other conditional treatments of carbide nanopowders. In this study, the effect of gamma irradiation on phase composition and microstructure of Cr_3_C_2_ nanopowders was examined for the first time. The results may be helpful to investigate the mechanism of the interaction between gamma ray and carbide materials.

## 2. Results and Discussion

**Figure 1:**
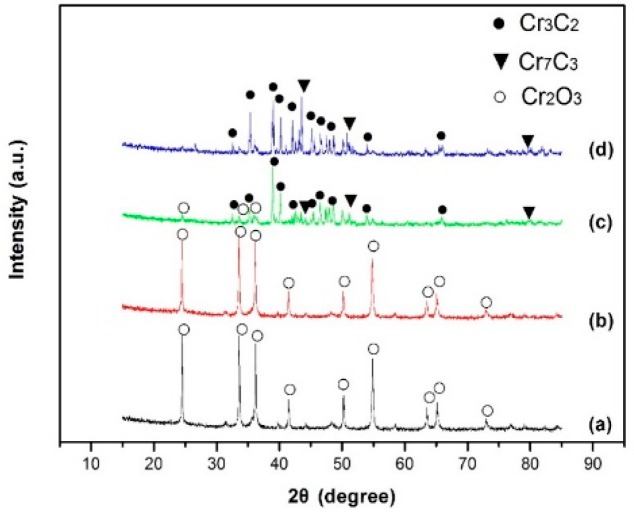
XRD patterns of the powders before and after microwave heating: (**a**) before microwave heating (32 wt% C); (**b**) before microwave heating (34 wt% C); (**c**) 1000 °C, 1 h (32 wt% C); (**d**) 1000 °C, 1 h (34 wt% C).

Figure 1 shows the XRD patterns of the powders after mechanical alloying and their microwave synthesis products at 1000 °C for different carbon contents (32 wt% C and 34 wt% C). As shown in Figure 1a,b, all peaks were identified as Cr_2_O_3_, while carbon black existed in amorphous form. Furthermore, there were clear signs of peaks broadening, indicating that the particle size of the raw materials became smaller after being milled by the planetary ball mill [23]. This shows that ball milling for a long time did not affect particle composition, while reducing the material diameter. From Figure 1c,d, it can be seen that the product was composed of Cr_3_C_2_ (JCPDS 35-0804), Cr_7_C_3_ (JCPDS 11-0550), and Cr_2_O_3_ (JCPDS 38-1479). This indicated that the oxidation-reduction reaction between Cr_2_O_3_ and carbon was not completed when the carbon content was 32 wt% C. With the increasing of the carbon content, as shown in Figure 1d, the product was mainly composed of Cr_3_C_2_ (JCPDS 35-0804) and Cr_7_C_3_ (JCPDS 11-0550), indicating that the oxidation-reduction reaction between Cr_2_O_3_ and carbon was completed when the carbon content was 34 wt% C.

**Figure 2:**
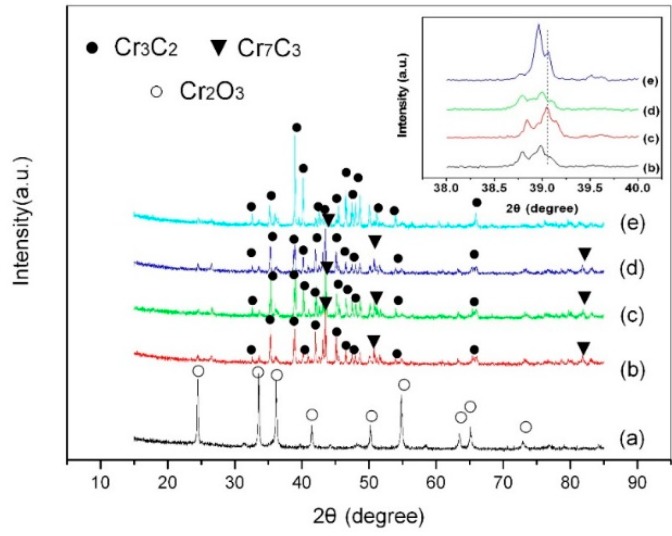
XRD patterns of the powders before and after microwave heating: (**a**) before microwave heating; (**b**) 0.5 h; (**c**) 1 h; (**d**) 1.5 h; (**e**) 2 h. The insertion is an enlargement of the diffraction peaks of the products at 38–40°.

Figure 2 shows the XRD patterns of the powders after mechanical alloying and their microwave synthesis products at 1000 °C for different holding time (0.5 h, 1 h, 1.5 h, and 2 h). The insertion is an enlargement of the diffraction peaks of the products at 38–40°. As shown in Figure 2a, all peaks were identified as Cr_2_O_3_ without the carbon phase. In Figure 2b, it can be seen that the product was mainly composed of Cr_3_C_2_ (JCPDS 35-0804) and Cr_7_C_3_ (JCPDS 11-0550), indicating that the oxidation-reduction reaction between Cr_2_O_3_ and carbon was completed at 1000 °C for 0.5 h. With the increase of the holding time, the intensity of the diffraction peaks of Cr_7_C_3_ decreased gradually, while Cr_3_C_2_ it showed the opposite trend (Figure 2c,d). When the holding time reached 2 h, all peaks were identified as Cr_3_C_2_ (Figure 2e), indicating that Cr_7_C_3_ (JCPDS 11-0550) completely transformed into Cr_3_C_2_ (JCPDS 35-0804). In the insertion in Figure 2b,c, it can be seen that the diffraction peaks of Cr_3_C_2_ shifted toward larger angles with the increase of the holding time, which was caused by the crystallization of chromium carbide. As shown in the insertion in Figure 2c–e, the diffraction peaks of Cr_3_C_2_ shifted toward smaller angles with a prolonged holding time. This happened mainly because the crystalline interplanar spacing of Cr_3_C_2_ increased with the increase of the holding time, which led to the decrease of the diffraction angle [23]. As mentioned above, a Cr_3_C_2_ phase with good crystallinity could be synthesized at 1000 °C for 1 h. The synthesis temperature required by the method here described was at least 400 °C lower than that of the conventional method (1400 °C) [5]. Furthermore, the synthesis temperature was 100 °C lower than those of the precursor and carbon thermal reduction methods [24,25].

**Figure 3:**
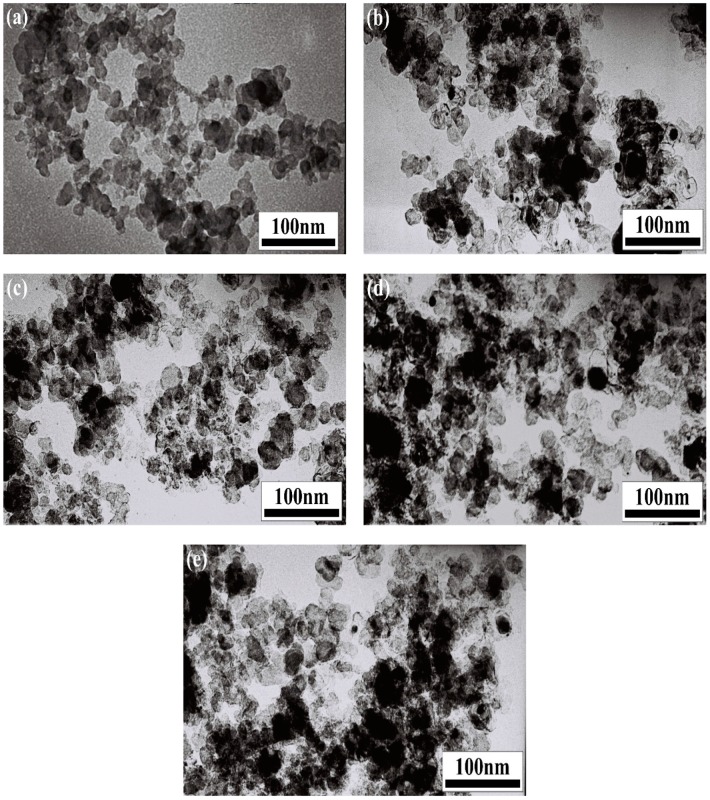
TEM micrographs of the powders before and after microwave heating: (**a**) before microwave heating; (**b**) 0.5 h; (**c**) 1 h; (**d**) 1.5 h; (**e**) 2 h.

Figure 3 shows typical TEM micrographs of the powders after mechanical alloying and their microwave synthesis products at 1000 °C for different holding time (0.5 h, 1 h, 1.5 h, and 2 h).

As shown in Figure 3a, the powders after mechanical alloying showed good dispersion and were mainly composed of particles with a mean diameter of about 50 nm. This was mainly because the particles were constantly impacted and fractured, leading to a considerable reduction of the particle size as a result of the energy provided during ball milling. However, slight agglomeration occurred in the powders as a result of intense mechanical deformation, refinement, and cold welding of particles during mechanical alloying [10,26]. When the holding time was 0.5 h, most powders showed good dispersion, and a small amount of particles exhibited the aggregation phenomenon, which might have been caused by the incomplete crystallization of Cr_3_C_2_, as shown in Figure 3b. When the holding time reached 1 h, the powders showed good dispersion and were mainly composed of spherical or nearly spherical particles with a mean diameter of about 50 nm, as shown in Figure 3c. When the holding time reached 1.5 h and 2 h, the powders exhibited many agglomerated particles (Figure 3d,e), indicating that longer holding times led to the occurrence of molten particles [25].

**Figure 4:**
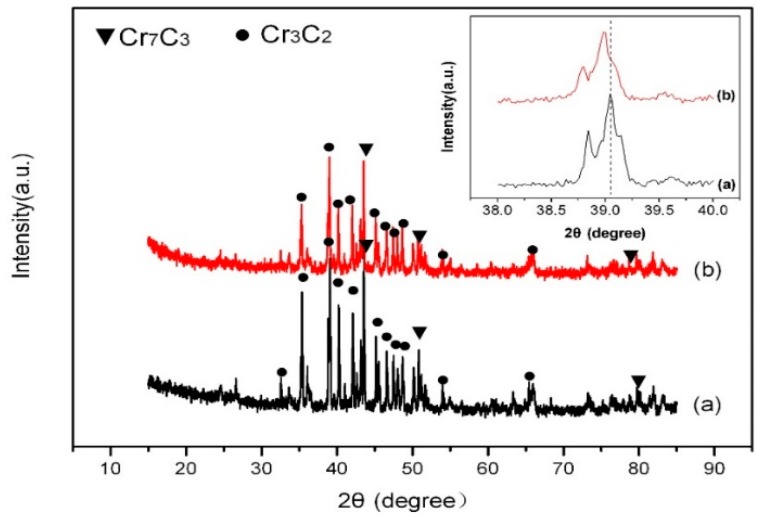
XRD patterns of the powders (1000 °C, 1 h) before and after gamma ray irradiation: (**a**) before gamma ray irradiation; (**b**) after gamma ray irradiation. The insertion is an enlargement of the diffraction peaks of the products at 38–40°.

**Figure 5:**
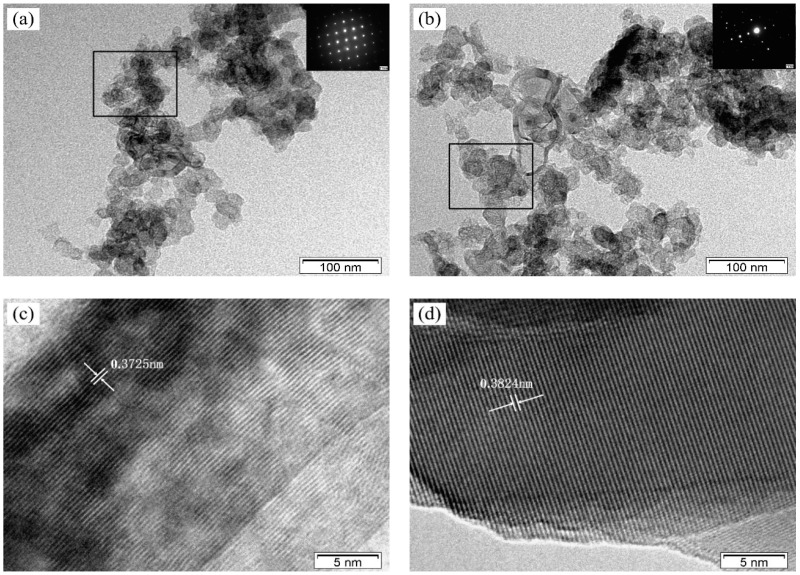
(**a**) TEM image of the sample before gamma ray irradiation; the inset shows the corresponding SAED pattern; (**b**) TEM image of the sample after gamma ray irradiation; the inset shows the corresponding SAED pattern; (**c**) HRTEM image of the sample before gamma ray irradiation; (**d**) HRTEM image of the sample after gamma ray irradiation.

**Figure 6:**
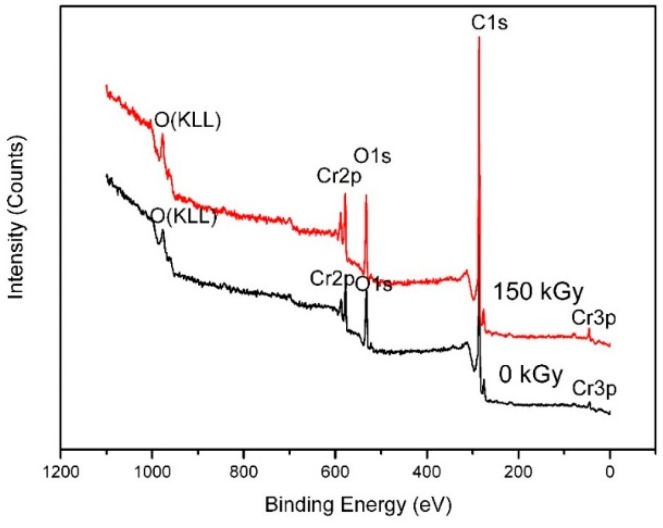
XPS spectra of the sample (1000 °C, 1 h) before and after gamma ray irradiation.

**Figure 7:**
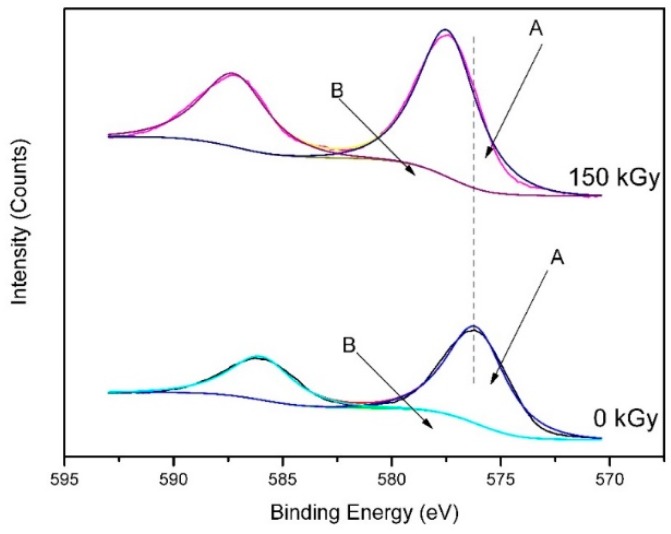
XPS spectrum of the Cr2p energy region for the powders (1000 °C, 1 h) before and after gamma ray irradiation.

To determine the effect of gamma irradiation on the microstructure of the synthesized nanopowders, XRD, TEM, and XPS measurements were carried out on the samples prepared by the microwave heating method at 1000 °C for 1 h in argon gas atmosphere, as shown in Figure 4, Figure 5, Figure 6 and Figure 7. Figure 4 shows the XRD patterns of the powders (1000 °C, 1 h) before and after gamma ray irradiation. The insertion in Figure 4 is an enlargement of the diffraction peaks of the products at 38–40°. As shown in Figure 4, the products were mainly composed of Cr_3_C_2_ (JCPDS 35-0804) and Cr_7_C_3_ (JCPDS 11-0550), indicating that the phase compositions of the samples before and after gamma irradiation did not changed. According to the Scherrer equation [23], the average crystallite sizes of the powders before and after gamma irradiation were 26 nm and 35 nm, respectively. This indicates that gamma irradiation can promote the growth of carbide grains. Comparing the peaks in the insertion in Figure 4, it can be seen that the diffraction peaks of Cr_3_C_2_ after irradiation with 150 kGy gamma ray shifted toward smaller angles. According to the Bragg equation 2*d*sin*θ* = *nλ* (*d* is the crystalline interplanar spacing, *θ* is the Bragg angle, *n* is an integer, *λ* is the wavelength of a beam of X-rays incident on a crystal with lattice planes separated by the distance *d*), the diffraction angle decreased with the increase of crystalline interplanar spacing. Furthermore, the diffraction peaks of irradiated Cr_3_C_2_ had a lower intensity than the peaks of the unirradiated sample. These phenomena are proposed to be attributed to the conversion of gamma radiation energy to crystal lattice energy and the entry of a small amount of carbon atoms into the Cr_3_C_2_ lattice, which led to the increase of the crystalline interplanar spacing and the change of crystal structure of Cr_3_C_2_ [27]. They may also be due to the transformation of Cr^3+^ with small ionic radium (0.61 nm) into Cr^2+^ with larger ionic radium (0.73 nm) during the reduction of a small amount of residual Cr_2_O_3_ to Cr_3_C_2_. Similar phenomena have been observed in the literature [28,29].

Figure 5 shows the TEM and HRTEM images of the samples before and after gamma ray irradiation. As shown in Figure 5a, the powders before gamma ray irradiation showed good dispersion and were mainly composed of particles with a mean diameter of about 50 nm. The selected area’s electron diffraction (SAED) pattern in Figure 5a clearly shows the presence of diffraction dots, which indicates that the selected area presented a single-crystal nature. However, as shown in Figure 5c some of the atoms were arranged irregularly. This was mainly due to the fact that Cr_3_C_2_ (JCPDS 35-0804) is a substoichiometric carbide [30]; it belongs to the orthorhombic system with ordered C vacancies, and its lattice parameters are 0.5527 nm × 1.1488 nm × 0.2829 nm. The interplanar spacing in Figure 5c was calculated and resulted to be 0.3725 nm, which is consistent with that of a (120) plane (d = 0.3704 nm) of Cr_3_C_2_ (JCPDS 35-0804). Compared with Figure 5a, the powders after gamma ray irradiation showed obvious aggregation, and the particles appeared larger (Figure 5b). Furthermore, the SAED pattern in Figure 5b shows that the diffraction spots were irregularly distributed, and the crystal structure of Cr_3_C_2_ appeared changed, which was mainly caused by a small amount of carbon atoms entering empty spaces or to the transformation of small-radium ions to large-radium ions during the reduction of Cr_2_O_3_ to Cr_3_C_2_. These results are consistent with the XRD measurements (Figure 4). The interplanar spacing in Figure 5d was calculated and the result was 0.3824 nm, which is obviously higher than that obtained before gamma ray irradiation. Besides, the arrangement of atoms in Figure 5d was more regular than that observed of Figure 5c. These phenomena are consistent with the results of SAED.

Figure 6 shows the XPS spectra of the samples (1000 °C, 1 h) before and after gamma irradiation. As shown in Figure 6, the surface of the specimen was mainly composed of Cr, C, and O. The peaks of A (576.1 eV→577.4 eV) and B (577.6 eV→587.3 eV) in Figure 7 were assigned to the Cr2p_3/2_ species of Cr_3_C_2−x_ (0 ≤ x ≤ 0.5) and Cr_2_O_3_, respectively. Furthermore, the peaks of the irradiated sample shifted toward higher binding energy and became sharper compared to those of the unirradiated powders. These phenomena may be caused by the diffusion of carbon atoms from the surface to the interior after gamma irradiation, leading to the increase of the crystalline interplanar spacing and the change of the crystal structure of Cr_3_C_2_ [31]. These results are consistent with the XRD and TEM measurements (Figure 4 and Figure 5). The higher binding energy improves the thermal stability and mechanical properties of Cr_3_C_2_ at high temperature.

## 3. Materials and Methods

**Figure 8:**
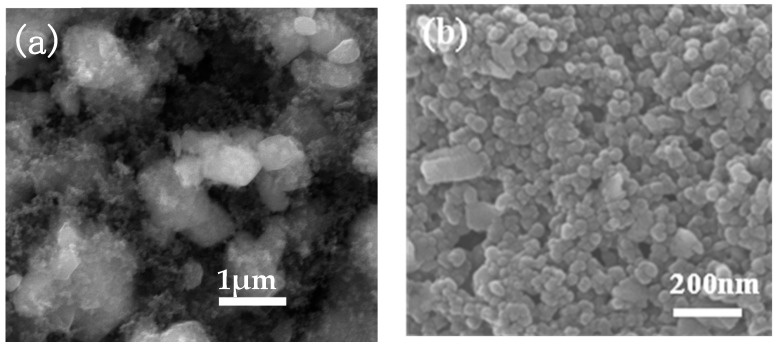
SEM micrographs of the powders obtained before and after mechanical alloying: (**a**) before mechanical alloying; (**b**) after mechanical alloying.

Micron-sized chromic oxide (Cr_2_O_3_) and nano-sized carbon black were used as raw materials. We put 66 wt% (68 wt%) Cr_2_O_3_ and 34 wt% (32 wt%) C into a QM-3SP2 high-energy planetary ball mill (Nanjing Lai Technology Industrial Co., Ltd., Jiangsu, China). The mixing and milling medium were absolute alcohol and cemented carbide balls, respectively. After milling for 64 h, the mixture was dried in a vacuum drying oven at 90 °C for 12 h. Figure 8 shows SEM micrographs of the powders obtained before and after mechanical alloying (34 wt% C). The powders before mechanical alloying were mainly composed of 1–2 μm and nano-sized particles (Figure 8a). The powders after mechanical alloying showed good dispersion and were mainly composed of spherical or nearly spherical particles with a mean diameter of about 100 nm (Figure 8b). Finally, the mixture was heated at 1000 °C for different holding time (0.5 h, 1 h, 1.5 h, and 2 h) at a heating rate of 15–50 °C/min in a multimode 2.45 GHz RWS microwave furnace (Zhongsheng Thermal Technology Co., Ltd., Hunan, China) in argon gas atmosphere to prepare Cr_3_C_2_ nanopowders. During microwave heating, 900–1100 W power was applied to the samples, and the temperature was measured by infrared measurement (Raytek Inc., Santa Cruz, CA, USA).

The samples were irradiated under gamma (^60^Co source) radiation in a radiation chamber with a dose rate of 100 Gy/min at an absorbed dose of 150 kGy. The crystal structure of the samples was analyzed by X-ray diffraction (XRD) using a Bruker D8 Advance diffractometer ( Bruker AXS Inc., Karlsruhe, Germany) with Cu-K_α_ radiation in the range of 2θ = 15 to 85°. X-ray photoelectron spectroscopy (XPS) was carried out using a XSAM 800 spectrometer (Kratos Analytical, Manchester, UK) with MgK (α 1) X-ray source. The morphology and microstructure of the samples were examined by JSM-6700F scanning electron microscopy (SEM), JEM-1000CX, and JEM-2100 transmission electron microscopy (TEM) (JEOL Ltd., Tokyo, Japan).

## 4. Conclusions

Cr_3_C_2_ nanopowders were synthesized via mechanical alloying and subsequent microwave heating. The powders prepared at 1000 °C for 1 h showed good dispersion and were mainly composed of spherical or nearly spherical particles with a mean diameter of about 50 nm. The synthesis temperature required by the presented method was at least 400 °C lower than that of conventional methods. Shorter holding time led to the appearance of oxides and agglomeration. On the contrary, longer holding time led to the increase of crystalline interplanar spacing of the carbide and the occurrence of molten particles. Gamma ray irradiation had an important effect on the microstructure of Cr_3_C_2_ nanopowders. XRD, HRTEM, and XPS results showed that gamma ray irradiation can increase the crystalline interplanar spacing and the binding energy of Cr_3_C_2_, which improves the thermal stability and mechanical properties of Cr_3_C_2_ at high temperature. Besides, gamma ray irradiation can also lead to the increase of the crystalline interplanar spacing and the change of the crystal structure of Cr_3_C_2_.
